# Studies on Chromatographic Fingerprint and Fingerprinting Profile-Efficacy Relationship of Polygoni Perfoliati Herba

**DOI:** 10.1155/2013/693439

**Published:** 2013-08-19

**Authors:** Li Tian, Yang Zhao, Xin Zhou, Hua-Guo Chen, Chao Zhao, Xiao-Jian Gong

**Affiliations:** ^1^The Research Center for Quality Control of Natural Medicine, Guizhou Normal University, Guiyang 550001, China; ^2^Key Laboratory for Information System of Mountainous Areas and Protection of Ecological Environment, Guizhou Normal University, Guiyang 550001, China

## Abstract

Polygoni Perfoliati Herba is widely used in China with antibacterium, anti-inflammatory, expectorant, antitumor, and antivirus activities. To reveal the mechanisms of the activities of Polygoni Perfoliati Herba, the relationship between the fingerprinting profile and its bioactivities was investigated. In the present study, high-performance liquid chromatographic (HPLC) fingerprinting method was developed. The established method was applied to analyze 51 batches of Polygoni Perfoliati Herba samples collected from different locations or in different harvesting times in China. Chemometrics, including similarity analysis, hierarchical clustering analysis, and principal component analysis, were used to express their similarities. It was found that similarity values of the samples were in the range of 0.432–0.998. The results of analgesic tests indicated that Polygoni Perfoliati Herba could significantly inhibit pain induced by hot plate and acetic acid in mice. The results of anti-inflammatory tests showed that Polygoni Perfoliati Herba had good anti-inflammatory effects (*P* < 0.01) in two models including dimethyl benzene-induced ear edema and acetic acid-induced peritoneal permeability in mice. Combining the results from chromatographic fingerprints with those from bioactivities, we found that seven peaks from Polygoni Perfoliati Herba were mainly responsible for analgesic and anti-inflammatory activities.

## 1. Introduction

Polygoni Perfoliati Herba (PPH), the aerial part of *Polygonum perfoliatum *L., called “Gangbangui” in Chinese, is one of the traditional Chinese medicines (TCMs) in plant family Polygonaceae* Polygonum* [1]. It grows widely in Guizhou, Sichuan, Hunan, Guangxi, and others. Previous studies demonstrated that flavonoids [2–7], anthraquinones [4, 8], phenylpropanoids [8–10], terpenoids [8], and volatile oils [11] were major chemical components of PPH. PPH was reported to have antibacterium [12, 13], anti-inflammatory [12, 14], expectorant, antitumor [15, 16], and antivirus [14, 17] activities. As one of the “*Miao* medicines,” PPH is often used in Guizhou province and has been acknowledged by local people for the treatments of cough, fever, and food poisoning [1]. Recently, it has been used in more and more formulas as the main herb, especially in some preparations for the treatments of gynecological inflammation, such as “Kangfuyan” capsule, “Fuping” capsule, and “Fuyanxiao” capsule. However, basic researches in quality control of PPH were considerably deficient; only a few quantification methods have been reported [18–22].

PPH has been used as the key raw material in formulas in many pharmaceutical companies, but, due to the lack of its basic researches, the active ingredients and the therapeutic mechanisms of PPH are still unclear so far.

In this study, it is aspired to (1) develop a chromatographic fingerprinting method of PPH, (2) analyze the fifty-one batches of PPH samples from different regions of China and find the variations of the chemical profiles, (3) investigate analgesic and anti-inflammatory activities of the selected twenty-five PPH samples, and (4) indicate and identify peaks potentially responsible for the activities obtained from different models.

## 2. Materials and Methods

### 2.1. Plant Materials and Reagents

A total of 51 batches of PPH samples were collected from different herbal markets or harvested from various regions in China, the information was summarized in Table 1. These samples were deposited in the Research Center for Quality Control of Nature Medicine, Guizhou Normal University. 25 batches of them were used in the present study for exploration on bioactivities and profile-effect correlations.

Analytical-grade ethanol (Fuyu Co., Tianjin, China) was used for sample preparation. LC-grade methanol (TEDIA, Company Inc., USA) was used to dissolve the extracts. Analytical-grade phosphoric acid (Kelong Co., Chengdu, China), LC-grade acetonitrile (TEDIA, Company Inc., USA), and purified water were used as mobile phases. Evans blue (Solarbio Co., Peking) and dimethyl benzene (Chuandong Chemical Co., Chongqing, China) were of analytical grade used for pharmacological experiments.

### 2.2. Sample Preparation

Each batch of dried PPH raw material was ground into powder (50 meshes).

1. For HPLC analysis, 1.0 g of each powder was accurately weighed into a 150 mL conical flask and extracted with 80 mL of 70% ethanol for 3 h at 80°C using a Soxhlet apparatus. The extracted solution was filtered through analytical paper. Then the filtered solution was evaporated to dryness and the residue dissolved in 25 mL of methanol. The supernatant was filtrated through a 0.45 *μ*m membrane for HPLC analysis.
2. For animal tests, 120 g of each powder was accurately weighed into a 2 L volumetric flask and extracted with 1.2 L of 70% ethanol according to extraction method mentioned previously. The filtered solution was evaporated to dryness. Each extract of PPH was dissolved in wide mouthed bottle using distilled water for animal tests.
3. For HPLC analysis of 25 samples of fingerprinting profile efficacy, according to dry extract yield of these samples, dry extract of each sample was accurately weighed into 25 mL volumetric flask and dissolved in 25 mL of methanol. The supernatant was filtrated through a 0.45 *μ*m membrane for HPLC analysis.

### 2.3. Instrumentation and Chromatographic Conditions

HPLC fingerprint analysis was carried out on an Agilent 1100 liquid chromatography, equipped with vacuum degasser, a quaternary pump, and a diode array detector (DAD), an autosampler and a column temperature controller. Data collection was performed by using ChemStation software. Analysis was performed at 30°C on a reversed phase LiChrospher-C_18_ column (250 × 4.6 mm, 5 *μ*m; Jiangsu Hanbon Science and Technology Co. Ltd., China). The mobile phase consisted of 0.05% phosphoric acid aqueous solution (A) and acetonitrile (B) eluted as the following program for separation: 0–10 min, 10%–15% B; 10–40 min, 15%–25% B; 40–55 min, 25%–35% B; 55–75 min, 35%–50% B; 75-76 min, 50%–90% B; 76–85 min, 90% B. The flow rate was 1 mL/min. The detection wavelength was set at 340 nm. An aliquot of 20 *μ*L solution was injected for HPLC fingerprinting analysis.

The U-HPLC/HRMS system consisted of an LTQ Orbitrap XL mass spectrometer with an Accela 1250 binary pump, a PAL HTC Accela TMO autosampler, and an Accela PDA detector (Thermo Fisher Scientific, San Jose, CA, USA). The column and elution conditions used were the same as those used in HPLC analysis except that the flow rate was set at 0.25 mL/min with a split ratio. The MS conditions were set as follows: sheath gas at 35 (arbitrary units), aux and sweep gas at 10 (arbitrary units), spray voltage at +3.5 KV/−3.0 KV, capillary temperature at 300°C, and heater temperature at 300°C. The mass range was from 120 to 1500 *m/z* with a resolution of 60000.

### 2.4. Validation of HPLC Fingerprinting Method

Precision was assessed with repetitive injection of the same sample (sample no. 10) solution for six times per one day. Six independently prepared sample solutions of PPH (sample no. 10) were analyzed for repeatability evaluation. The stability test was determined with sample no. 10 analyzed at 0, 2, 4, 8, 12, and 24 h. Quercetin-3-O-*β*-D-glucuronide, a large single peak in the middle of the chromatograms of all the PPH samples, was assigned as the reference peak to calculate the relative retention time (RRT) and relative peak area (RPA). The *RSD* of RRT and RPA of common peaks were calculated for assessment of the established method.

### 2.5. Analyses of HPLC Fingerprints

#### 2.5.1. Similarity Analysis

Similarity Evaluation System (SAS) for Chromatographic Fingerprint of Traditional Chinese Medicine (version 2004 A) was recommended by Chinese Pharmacopoeia Commission and was used for similarity analysis of chromatographic fingerprints in the present study. Briefly, each chromatogram was exported from Agilent 1100 ChemStation software as *AIA format, which was then imported into SAS. The mean chromatogram was generated, and the similarity values were calculated after multipoint correction was performed.

#### 2.5.2. HCA

HCA is a multivariate analysis technique that is used to classify samples into groups. This technique comprehends an unsupervised classification procedure that involves measuring either the distance or the similarity between the objects to be clustered. In the present study, SPSS (version 13.0) was used to analyze the samples, using PA/W values (peak areas of the common peaks divided by sample weight) as variables. Ward's method, which is very efficient for the analysis of variances between clusters, was applied, and square Euclidean distance was selected as a measurement.

#### 2.5.3. PCA

PCA is a sophisticated technique widely used for reducing the dimensions of multivariate problems. It involves a mathematical procedure that transforms a number of possible correlated variables into a smaller number of uncorrelated variables called principle components (PCs) [23–25]. In the present study, SIMCA-P 11.5 (Umetrics AB, Umea, Sweden) was used for PCA after the mean centering and unit variance (UV) scaling were accomplished, using PA/W values as variables.

### 2.6. Selection of PPH Samples for Animal Tests

Based on the chromatographic fingerprints, those samples with significant variations in chemical profiles were selected to investigate their analgesic and anti-inflammatory bioactivities as well as profile-effect correlations.

### 2.7. Analgesic and Anti-Inflammatory Activities of PPH

#### 2.7.1. Animals

Female and male Kunming mice (20 ± 2 g) used in the present experiments were purchased from the Animal Laboratories Technique Department of Dongchuang (Changsha, China). The experimental animals were randomly divided into many groups, and each group had ten. They were housed in standard cages at a constant temperature of 20 ± 2°C with 12 h dark-light cycle for at least one week before the experiments. The mice were fed with food and water ad libitum. Animal tests were performed according to “Principles of Laboratory Animal Care and Use in Research” (Ministry of Health, Beijing, China).

#### 2.7.2. Oral Administration of PPH

Mice were orally administered with three doses (1400 mg/kg, 2800 mg/kg, and 5600 mg/kg) of ethanol extracts of the selected 25 batches of PPH (shown in Table 2) and aspirin (300 mg/kg, reference drug; Pingguang Pharmacy of Jiangsu, China) once a day, respectively. The mice in control group were given the same volume of distilled water. The doses for oral administration were confirmed according to the method described in the literature [26] and were suitable for the study.

#### 2.7.3. Hot Plate Test

Hot plate test was performed according to the previously described methodology [27]. Briefly, mice were placed on a hot plate maintained at 55 ± 0.5°C. The time that elapsed until occurrence of either a hind paw licking or a jump off the surface was recorded as the hot plate latency. Mice with baseline latencies of >30 s or <5 s were excluded from the study. After the determination of baseline response latencies, hot plate latencies were measured at 15, 30, 60, and 90 minutes after the last administration of the extracts of the 25 batches of PPH, aspirin, and distilled water. If mice did not lick their hind paws within 60 s, 60 s would be recorded as the value. For each group, the percentages of pain inhibition were calculated according to the following formula: pain inhibition percentage (PIP) = (*T*1 − *T*0) × 100/*T*0, where *T*1 is latency after drug and *T*0 is latency before drug. Differences in pre- and post-drug latencies were analyzed by Student's *t*-test.

#### 2.7.4. Acetic Acid-Induced Writhing Test

Acetic acid-induced writhing test was performed according to the literature [28]. The mice were injected i.p. with 0.1 mL/10 g of 0.6% acetic acid 30 min after the last oral administration of the ethanol extracts of PPH and aspirin. Control animals received distilled water under the same experimental condition. The number of writhing reflexes was counted during the following 15 min.

#### 2.7.5. Dimethyl Benzene-Induced Mice Ear Edema Test

The anti-inflammatory effect of PPH was investigated in acute inflammation method [29] with some modifications. On the 8th day, 45 min after mice were orally administered with the drugs, the right ear of each rat was applied with dimethyl benzene (40 *μ*L) on both ear surfaces, and the left ear was used as the control. The animals were executed by cervical dislocation after 15 min. A 6 mm section from each ear was moved with a metal punch and weighed. The edema weight and inhibition percentage are evaluated according to the following equations:

(1) edema  weight=weight  of  the  right  ear −weight  of  the  left  ear,inhibition% T=(( edema weight of control group  − edema weight of the drug groups)  × (edema  weight  of  control)−1)×100%.

#### 2.7.6. Acetic Acid-Induced Vascular Permeability in Mice

This test was performed using the method described by Whittle [30] with some modifications. Briefly, the mice received PPH extracts, aspirin, and distilled water once a day for 7 days. Half an hour after the last treatment, each mouse was injected with 0.2 mL of 2% Evan's Blue in normal saline solution intravenously through the tail. Thirty minutes later, each mouse was injected intraperitoneally with 0.2 mL of 0.6% acetic acid (v/v). Twenty minutes after intraperitoneal injection later, the mice were killed, and the peritoneal cavities were exposed and washed with 5 mL of normal saline to collect pigments in a test tube. After being centrifuged, the absorbance of each supernatant was measured at 590 nm using an ultraviolet spectrophotometer.

#### 2.7.7. Statistical Analysis

Comparison between treated groups and control groups was carried out by ANOVA and Student's *t*-tests. *P* < 0.05 was considered to be different significantly.

## 3. Results and Discussion

### 3.1. Optimization of Extraction Conditions

Selection of an extraction strategy plays an important role for the fingerprint analysis of TCM on account of its complexity. In order to develop an optimum extraction process, heating reflux extraction and ultrasonic extraction methods were evaluated. As a result, stronger signals of the characteristic peaks were obtained using reflux extraction, which was then selected as the extraction method.

With regard to extraction solvents, methanol, ethanol, and ethyl acetate were tested in the present study, and the results indicated that ethanol was the most preferred one. Then, different concentrations of ethanol solution (60%, 70%, 80%, and 95%) were tested. The results showed that 70% aqueous ethanol was the preferred one which gave the most abundant fingerprinting information. PPH powder (1 g) was extracted with 70% ethanol for 1, 2, 3, and 3.5 h, respectively. It was found that peak areas of the reference compounds did not increase significantly any more (*P* < 0.05) after the sample was extracted for 3 hours, which was then set as the best extraction time. Based on the previous results, the extraction times and solvent volume were further optimized; as a result, extraction method was finalized, as stated in “sample preparation.”

### 3.2. Optimization of HPLC Conditions

To establish an ideal fingerprint, the decisive chromatographic parameters including columns, mobile phases, flow rate of mobile phases, and detection wavelengths, were optimized. Different columns (ZORBAX SB-C_18_, Eclipse XDB-C_8_, and LiChrospher-C_18_) were tested in the present study before LiChrospher-C_18_ column (250 × 4.6 mm, 5 *μ*m) was finally selected as the column of choice. In order to gain better baseline, resolution, and peak shape, three kinds of acids, acetic acid, formic acid, and phosphoric acid, were investigate. The results demonstrated that the best baseline and the most satisfactory resolution of major peaks could be obtained using acetonitrile and 0.05% (v/v) phosphoric acid as mobile phases. DAD was used to acquire chromatograms from 190 to 400 nm; finally, 340 nm was selected as detection wavelength to get chromatographic fingerprints.

### 3.3. HPLC Fingerprint and Similarity Analysis

**Figure 1:**
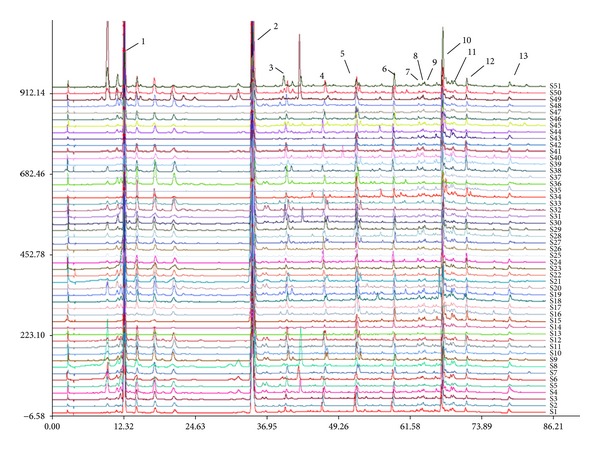
Overlapped HPLC chromatographic fingerprints of the 51 batches of PPH samples.

LC chromatographic fingerprints of 51 batches of PPH were used to get the “common pattern” using the professional software Similarity Evaluation System for Chromatographic Fingerprint of Traditional Chinese Medicine composed by China Pharmacopoeia Committee (version 2004 A). The overlapped chromatograms of PPH are shown in Figure 1. Thirteen common peaks were found within 85 min and shown in Figure 1. The RSDs of RRT and RPA of the thirteen common peaks are listed in Table 3.

Similarities of the PPH samples were calculated using mean fusion vector method; the results are listed in Table 4. These similarity values were in range of 0.432–0.998, which could be divided into three ranges, below 0.90 (7 batches), 0.90–0.99 (30 batches), and 0.99–1.00 (14 batches). Two meaningful phenomena were found from the similarity results. On one hand, some samples from the same region in different harvesting times differed a lot in their similarity values. For example, samples no. 8 and no. 9 were collected from Danzhai and Guizhou in August 2008 and October 2010, respectively, but their similarity values were 0.893 and 0.993, respectively. Meanwhile, samples no. 33 and no. 34 were both from Shuicheng and Guizhou, but they were harvested in August 2008 and August 2010, respectively, leading their similarities to 0.969 and 0.933, respectively. On the other hand, samples from the same city harvested in the similar period of time had similar values. For instance, samples nos. 25, 26, 27, 29, and 30 were all from Guiyang city collected in August 2008; their similarity values were 0.980, 0.991, 0.962, 0.992, and 0.988, respectively, indicating that these samples were similar in chemical profiles due to their similar growing environment and outer climate conditions.

### 3.4. Hierarchical Clustering Analysis

Hierarchical clustering analysis is one of the statistical systematic methods that can evaluate the resemblance, as well as the classification of different objects.

**Figure 2:**
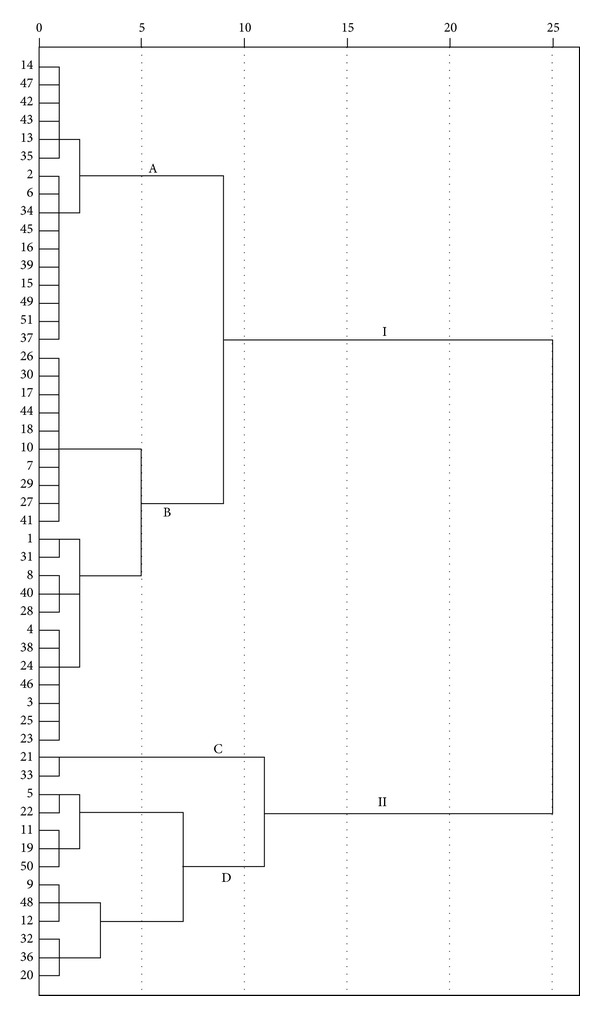
HCA results of the 51 batches of PPH samples based on the PA/W values of the thirteen common peaks. Ward's method was applied, and square Euclidean distance was selected as the measurement.

In the present study, 13 peaks which existed in all the chromatograms were selected as common peaks, which are showed in Figure 1. The relative peak areas of these common peaks were calculated, forming a 13 (peaks) × 51 (samples) matrix, before HCA. The matrix was performed by SPSS, which generated a dendrogram shown in Figure 2, indicating the similarities of the samples. The dendrogram showed clearly that the 51 tested samples were divided into two main clusters: I and II, which were further divided into four subgroups: A, B, C, and D. The samples with similar chemical profiles were clustered into the same subgroup. It was found that (1) samples from the same region were not clustered into one group, but those with similar RPA values of common peaks were classified into the same group; (2) big peaks played more important roles than small ones in classification.

### 3.5. PCA

**Figure 3:**
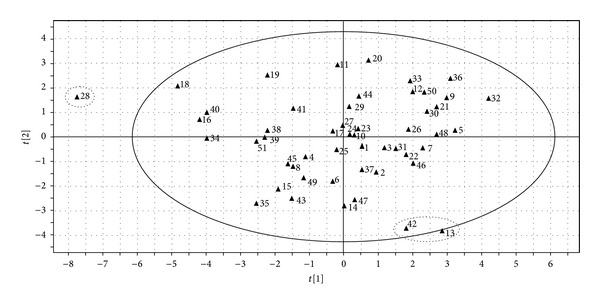
PCA scores plot of the 51 batches of PPH samples. PA/W values of the thirteen common peaks were used as input data.

**Figure 4:**
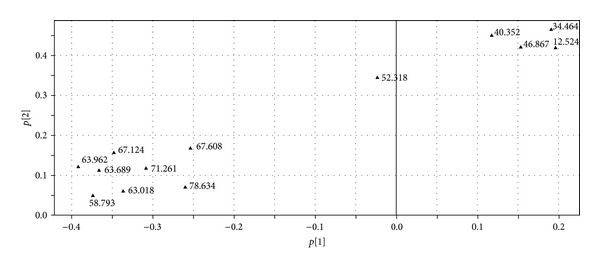
PCA loadings plot of the 51 batches of PPH samples. *p*[1] and *p*[2] represent the first and the second principal components, respectively.

Principal component analysis is a method of multiple statistical analysis applying the degradative ideal to transformed multicriteria to a few synthetic index; it uses fewer variable to explain the most variables of aboriginal data. In the present study, RPA of the 13 common peaks were used as the variables for PCA in SIMCA-P software to analyze the similarities of the 51 batches of samples. The scores plot obtained from PCA is shown in Figure 3. It is shown that the samples mainly distribute into three domains; sample no. 28 can be regarded as an outlier which locates outside the ellipse (95% confidence interval); samples no. 42 and no. 13 distribute closely, while the others are in a big domain. To find the peaks which were responsible for the distribution of the samples in scores plot, loadings plot (Figure 4) was generated. The loadings plot indicated that two peaks at retention time of 12.524 min (phenolic compounds) and 34.464 min (quercetin-3-O-*β*-D-glucuronide) might have the most significant influence on the classification of the samples than other peaks, and they might be regarded as the chemical markers for quality control on different PPH.

### 3.6. Selection of Representative Samples for Bioactive Experiments

On the basis of chromatographic fingerprints of the 51 batches of PPH and the chemometrics including similarity evaluation, PCA, and HCA, 25 batches of PPH with different chemical profiles were selected for researches on their activities and for profile-efficiency study.

### 3.7. Analgesic and Anti-Inflammatory Activities of PPH

#### 3.7.1. Hot Plate Test

**Figure 5:**
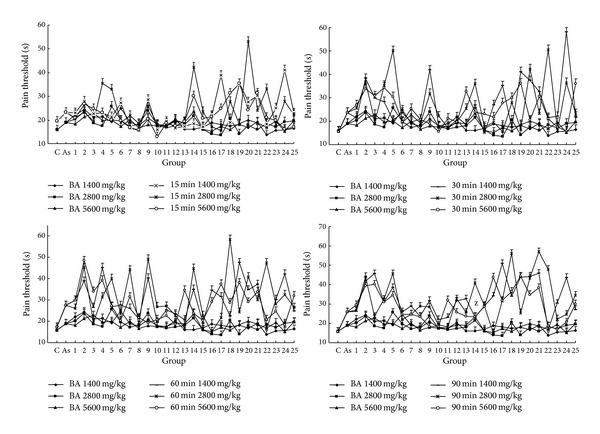
The results of hot plate test in mice. Each value represents mean ± SD. BA: before administration.

The results of hot plate test are presented in Figure 5 (C and As represent the control group and the aspirin group, resp.); it indicated that the 25 batches of PPH samples had distinct effects on pain compared with control group and aspirin group (*P* < 0.01). The pain thresholds of the PPH groups after administration were higher than those before administration (*P* < 0.01). The thresholds increased 15 ~ 90 min after the last administration compared with the ones in control group. The analgesic effects of samples 2, 4, 7, 9, 14, 17, 18, 19, 20, 21, and 24 were better than those of other groups. Sample no. 18 had the best analgesic activity when orally administrated at 2800 mg/kg in all samples. However, samples 1, 8, and 10 did not suppress the pain of mice induced by hot plate in the study.

#### 3.7.2. Writhing Test

**Figure 6:**
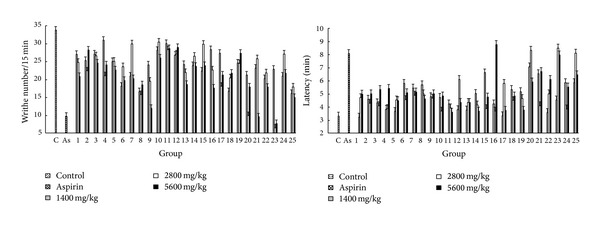
The results of writhing test in mice. Each value represents mean ± SD.

The results of acetic acid-induced writhing responses in mice were indicated in Figure 6. It was demonstrated that 25 batches of ethanol extracts of PPH at three doses (1400, 2800, and 5600 mg/kg) could significantly reduce the number of writhing and delay the response latency in comparison with the values obtained from control group (*P* < 0.01). The activity of sample no. 23 was the best at the dose of 2800 mg/kg, and the inhibition rate compared with the value from the control group was 80.74% (*P* < 0.01), which was higher than the one from aspirin group at the dose of 300 mg/kg (73.26%, *P* < 0.0001).

#### 3.7.3. Dimethyl-Benzene Induced Mice Ear Edema Test

**Figure 7:**
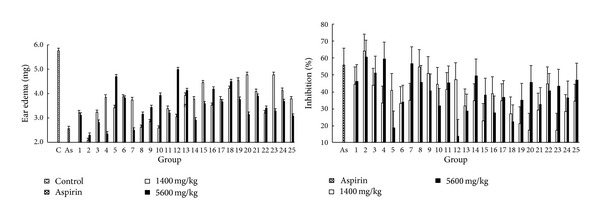
Anti-inflammatory effect of PPH extract on edema in mice induced by dimethyl benzene. Each value represents mean ± SD.

Dimethyl benzene-induced mice ear edema test is an experiment investigating anti-inflammatory activity. The results obtained from this model were presented in Figure 7. It was found that PPH significantly restrained the dimethyl benzene-induced ear edema at three doses compared with the values obtained from control group. Moreover, the inhibition rates of sample no. 2 were 63.91% (*P* < 0.01) and 60.22% (*P* < 0.01) at doses of 1400 and 5600 mg/kg, respectively, which were both better than the values from aspirin group (55.53%, *P* < 0.0001).

#### 3.7.4. Vascular Permeability Test in Mice

**Figure 8:**
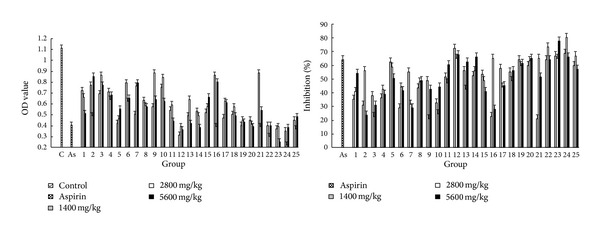
The results of vascular permeability test in mice. Each value represents mean ± SD.

Figure 8 shows the results of abdominal cavity vascular permeability test in mice, which shows that OD values of abdominal cavity lotion from PPH groups and aspirin group are lower than those from control group, indicating that the 25 batches of PPH samples significantly inhibited the acetic acid-induced vascular permeability in mice (*P* < 0.01). Aspirin is used as an anti-inflammatory drug to reduce acute inflammatory response; the inhibition rate of aspirin at the dose of 300 mg/kg was about 63.87% compared with the value obtained from control group. Samples nos. 12, 22, 23, and 24 had better inhibitory effects than aspirin at dose of 300 mg/kg. The inhibition of sample 24 was 80.18% at the dose of 2800 mg/kg, which was the highest one in all the samples.

### 3.8. Profile-Efficacy Study of PPH

**Figure 9:**
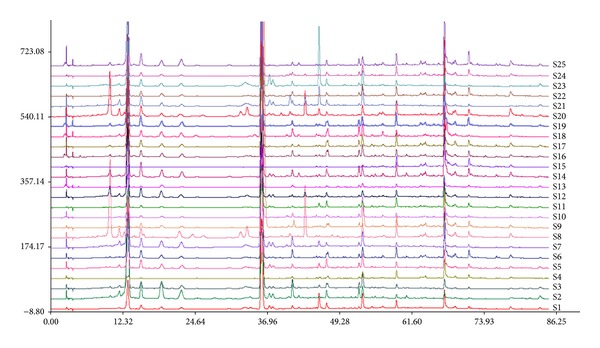
Overlapped HPLC chromatographic fingerprints of the 25 batches of PPH samples.

**Figure 10:**
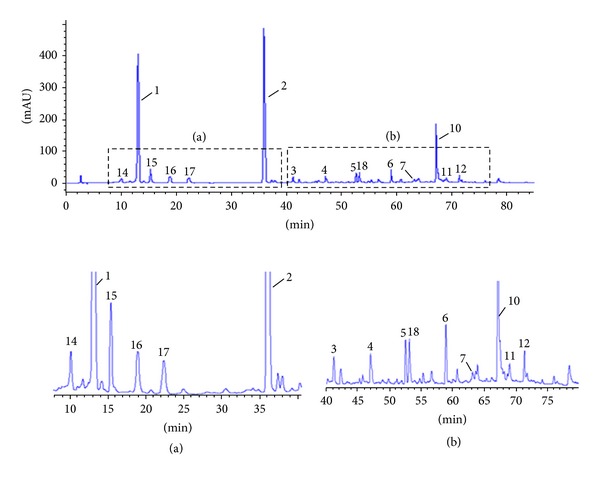
Representative chromatographic fingerprints obtained from sample no. 14. Fifteen characteristic peaks are marked. Assignments: peak 2, quercetin-3-O-**β**-D-glucuronide; peak 3, quercetin-3-O-**β**-D-glucuronide methyl ester; peak 4, ajugasterone C-20, 22-monoacetonide; peak 5, quercetin; peak 6, hydropiperoside; peak 7, vanicoside C; peak 10, vanicoside B; peak 11, vanicoside F; peak 16, parakmerin A; peak 17, O-hydroxycinnamoyl-**β**-D-glupyranoside; peak 18, helonioside B, respectively. (a) and (b) were enlarged views.

The 25 batches of PPH which were used for pharmacological studies were analyzed using the established fingerprinting method. Their overlapped fingerprinting chromatograms are shown in Figure 9. Fifteen characteristic peaks are marked and shown in Figure 10, among which peaks 2, 3, 4, 5, 6, 7, 10, 11, 16, 17, and 18 were identified as quercetin-3-O-**β**-D-glucuronide, quercetin-3-O-**β**-D-glucuronide methyl ester, ajugasterone C-20, 22-monoacetonide, quercetin, hydropiperoside, vanicoside C, vanicoside B, vanicoside F, parakmerin A, O-hydroxycinnamoyl-**β**-D-glupyranoside, and helonioside B, respectively, by comparing their retention times, UV spectra with standards, and some peaks were identified by comparing MS fragmentation with those of the reference compounds [7, 8, 31–36], which was shown in Table 5. Bivariate correlation analysis using SPSS (version 18.0) was performed to study the relationships between chemical profiles of the different fingerprints and the analgesic and anti-inflammatory activities of the 25 PPH samples.

#### 3.8.1. The Profile-Efficacy Relationship Obtained from Hot Plate Test

The correlation coefficients between the characteristic peaks in fingerprinting chromatograms and the analgesic efficacy in hot plate test at three doses of PPH are shown in Table 6. The results show that the correlation coefficients of peaks 1 and 15, parakmerin A, O-hydroxycinnamoyl-**β**-D-glupyranoside, quercetin-3-O*-*β**-D-glucuronide, quercetin-3-O-**β**-D-glucuronide methyl ester, and quercetin with the efficacies are higher than those from other peaks, indicating that the seven peaks are positively correlated with analgesic effect (*P* < 0.05).

#### 3.8.2. The Profile-Efficacy Relationship Obtained from Writhing Test

The correlation coefficients between the characteristic peaks in chromatographic fingerprints and the analgesic efficacy obtained from writhing test were shown in Table 7. It was found from the results that peaks 1 and 15, parakmerin A, O-hydroxycinnamoyl-**β**-D-glupyranoside, quercetin-3-O-**β**-D-glucuronide, quercetin-3-O-**β**-D-glucuronide methyl ester, and quercetin had positive relationships with analgesic activity in writhing test (*P* < 0.05).

#### 3.8.3. The Profile-Efficacy Relationship Obtained from Dimethyl Benzene-Induced Mice Ear Edema Test

Table 8 shows the relationship results between the characteristic peaks and the anti-inflammatory efficacy of PPH obtained from dimethyl benzene-induced mice ear edema test, indicating that peaks 1, 14, and 15, parakmerin A, O-hydroxycinnamoyl-**β**-D-glupyranoside, quercetin-3-O-**β**-D-glucuronide, quercetin-3-O-**β**-D-glucuronide methyl ester, and quercetin have higher correlation with the inhibition effect on dimethyl benzene-induced mice ear edema compared with other peaks (*P* < 0.05).

#### 3.8.4. The Profile-Efficacy Relationship Obtained from Vascular Permeability Test

The profile-efficacy relationship coefficients were analyzed and shown in Table 9, indicating that peaks 1 and 15, parakmerin A, O-hydroxycinnamoyl-**β**-D-glupyranoside, quercetin-3-O-**β**-D-glucuronide, quercetin-3-O-**β**-D-glucuronide methyl ester, and quercetin had positive correlations with the inhibition effect on anti-inflammation at three doses.

On the whole, with the aid of bivariate correlation analysis using SPSS software, the correlations of the characteristic peaks with the analgesic and anti-inflammatory activities of PPH were revealed. The research finding indicated that peaks 1 and 15, parakmerin A, O-hydroxycinnamoyl-**β**-D-glupyranoside, quercetin-3-O-**β**-D-glucuronide, quercetin-3-O-**β**-D-glucuronide methyl ester, and quercetin have good interrelationship with the analgesic and anti-inflammatory activities of PPH. Peak 1 was tentatively identified as one of the phenol compounds with the aid of ^1^H-NMR and ^13^C- NMR data to the best of our abilities.

## 4. Conclusions

In the present study, a simple, accurate, and validated chromatographic fingerprinting method was developed to analyze 51 batches of PPH samples collected from different regions in China. Thirteen peaks were designated as “common peaks” in chromatographic fingerprints of all the 51 PPH samples. Similarity analysis (SA), hierarchical clustering analysis (HCA), and principal component analysis (PCA) were further performed to analyze the data obtained from chromatographic fingerprints of the 51 PPH samples. The results indicated that the compounds with higher concentrations in raw PPH samples played more important roles than those with lower concentrations in classification. Then, 25 batches of samples with significant chemical diversities were selected in the present study for anti-inflammation and analgesic activities to reveal the relationships between fingerprinting profiles and activities of the herb. Fifteen peaks were marked as “characteristic peaks” in the 25 batches of samples, among which peaks 2, 3, 4, 5, 6, 7, 10, 11, 16, 17, and 18 were identified as quercetin-3-O-*β*-D-glucuronide, quercetin-3-O-*β*-D-glucuronide methyl ester, ajugasterone C-20, 22-monoacetonide, quercetin, hydropiperoside, vanicoside C, vanicoside B, vanicoside F, parakmerin A, O-hydroxycinnamoyl-**β**-D-glupyranoside, and helonioside B, respectively.

Anti-inflammation activity of PPH was evaluated with both dimethyl benzene-induced mouse inflammation model and acetic acid-induced mouse vascular permeability model. The analgesic activity of PPH was evaluated using hot plate test and acetic acid-induced writhing test. Experimental results indicated that the ethanol extract of PPH possessed good activities against inflammation and pain.

Bivariate correlation analysis was performed to reveal the relationships between fingerprinting profiles and pharmacological activities of PPH. The results indicated that eight peaks had positive correlations with analgesic activities; meanwhile, seven had positive correlations with anti-inflammatory activities.

## Figures and Tables

**Table 1:** The collected regions of tested Polygoni Perfoliati Herba samples.

Sample no.	Regions	Collection time
1	Duyun, GZ^a^	2008.08
2	Qiannan Normal College 1, GZ	2008.08
3	Qiannan Normal College 2, GZ	2008.08
4	Majiang, GZ	2008.08
5	Majiang, GZ	2010.10
6	Guiding, GZ	2008.08
7	Guiding, GZ	2010.07
8	Danzhai, GZ	2008.08
9	Danzhai, GZ	2010.10
10	Longli, GZ	2008.08
11	Longli, GZ	2010.07
12	Duyun, GZ	2010.10
13	Songtao, GZ	2010.08
14	Jiangkou, GZ	2008.08
15	Yanhe, GZ	2008.08
16	Songtao, GZ	2010.09
17	Huaguoyuan herb market, GZ	2011.03
18	Drug material market of Guiyang, GZ	2008.08
19	Zhazuo, Xiuwen, GZ	2010.08
20	Xiuwen, GZ	2010.08
21	Jiuchang, Xiuwen, GZ	2008.08
22	Xifeng, GZ	2010.07
23	Xifengshihuaping, GZ	2008.08
24	Xifeng, GZ	2008.08
25	Guangongpo,Guiyang, GZ	2008.08
26	Niulangguandaxingtian, Guiyang, GZ	2008.08
27	Huaxi, Guiyang, GZ	2008.06
28	Mengguantuba, Guiyang, GZ	2008.08
29	Xintianzhai, Guiyang, GZ	2008.08
30	Drug plant garden, GZ	2009.01
31	Guizun RD, Guiyang, GZ	2008.08
32	Guizun RD, Guiyang, GZ	2008.08
33	Shuicheng, GZ	2008.08
34	Shuicheng, GZ	2010.08
35	Liuzhi, GZ	2008.09
36	Chishui, GZ	2010.08
37	Renhuai, GZ	2010.08
38	Bijie, GZ	2008.08
39	Bijie, GZ	2010.09
40	Zhenfeng, GZ	2007.08
41	Yuancheng pharmaceutical company	2011.04 purchased
42	Tong Ren Tang	2011.04 purchased
43	Jinping, GZ	2008.09
44	Dafang, GZ	2010.08
45	Dabie Mountain, AH^b^	2008.08
46	Wenshu, HN^c^	2008.08
47	Daoxian, HN^d^	2010.05
48	Chun'an, ZJ^ e^	2008.08
49	Chenxi, HN	2008.08
50	Unknown 2	Unknown
51	Unknown 1	Unknown

^a^Guizhou province; ^b^Anhui province; ^c^Henan province; ^d^Hunan province; ^e^Zhejiang province.

**Table 2:** The 25 batches of PPH with big variations of chemical profiles.

Sample no.	Corresponding to the no. in fifty-one batches of PPH samples	Resources	Collected time
1	1	Duyun, GZ^a^	2008.08
2	36	Chishui, GZ	2010.08
3	17	Huaguoyuan herb market, GZ	2011.03
4	47	Daoxian, HN^b^	2010.05
5	46	Wenshu, HN^c^	2008.08
6	48	Chun'an, ZJ^d^	2008.08
7	40	Zhenfeng, GZ	2007.08
8	49	Chenxi, Hunan	2008.08
9	27	Huaxi, Guiyang, GZ	2008.06
10	37	Renhuai, GZ	2010.08
11	31	Guizun RD, Guiyang, GZ	2008.08
12	4	Majiang, GZ	2008.08
13	13	Songtao, GZ	2010.08
14	19	Xiuwen Zhazuo, GZ	2010.08
15	35	Liuzhi, GZ	2008.09
16	33	Shuicheng, GZ	2008.08
17	2	Qiannan Normal College, GZ	2008.08
18	44	Dafang, GZ	2010.08
19	28	Mengguantuba, Guiyang, GZ	2008.08
20	8	Danzhai, GZ	2008.08
21	30	Drug plant garden, GZ	2009.01
22	41	Yuancheng pharmaceutical company	2011.04, purchased
23	21	Jiuchang, Xiuwen, GZ	2008.08
24	45	Dabie Mountain, AH^e^	2008.08
25	38	Bijie, GZ	2008.08

^a^Guizhou province; ^b^Hunan province; ^c^Henan province; ^d^Zhejiang province; ^e^Anhui province.

**Table 3:** Retention time (RT), the RSD of relative peak areas (RPA), and RT of 13 common peaks in the fingerprint chromatograms.

Peak no.	RT	RSD (%) of RT	RSD (%) of RPA
1	12.5	1.009	54.089
2	34.4	0	0
3	40.3	0.897	63.129
4	46.9	0.703	51.237
5	52.3	0.745	73.613
6	58.8	0.775	165.488
7	63.0	0.791	174.120
8	63.7	0.679	141.659
9	64.0	0.777	158.522
10	67.1	0.702	146.595
11	67.6	0.783	140.519
12	71.3	0.768	172.458
13	78.6	0.765	135.824

**Table 4:** The similarity values of total samples.

Sample no.	Similarity	Sample no.	Similarity	Sample no.	Similarity
1	0.993	18	0.984	35	0.432
2	0.972	19	0.977	36	0.974
3	0.988	20	0.981	37	0.955
4	0.928	21	0.976	38	0.951
5	0.953	22	0.984	39	0.933
6	0.946	23	0.992	40	0.968
7	0.993	24	0.996	41	0.982
8	0.893	25	0.980	42	0.942
9	0.993	26	0.991	43	0.740
10	0.998	27	0.962	44	0.991
11	0.978	28	0.944	45	0.967
12	0.994	29	0.992	46	0.994
13	0.967	30	0.988	47	0.866
14	0.883	31	0.991	48	0.993
15	0.841	32	0.962	49	0.553
16	0.911	33	0.969	50	0.990
17	0.988	34	0.933	51	0.895

**Table 5:** MS fragmentation of the investigated compounds by HPLC-MS^a^.

Peak no.	RT (min)	UV *λ*_max⁡_ (nm)	PI/NI	Compound	References
14	11.8	330	283.17459 [M + H]^+^/—	Unknown	—
1	13.2	217, 326	330.08154 [M + NH_4_]^+^/311.04016 [M − H]^−^, 623.08777 [2M − H]^−^	Unknown	—
15	15.5	325	387. 20084 [M+ H]^+^/431.19141 [M + HCOOH-H]^−^	Unknown	—
16	19.0	315	319.04379 [M + Na]^+^/295.04529 [M−H]^−^, 591.09814 [2M−H]^−^	Parakmerin A	[32]
17	22.4	272, 328	—/325.05576 [M − H]^−^, 651.11853 [2M − H]^−^	O-Hydroxycinnamoyl-*β*-D-glupyranoside	[34]
2	36.0	258, 355	479.08060 [M + H]^+^/477.06668 [M − H]^−^, 955.14105 [2M − H]^−^	Quercetin-3-O-*β*-D-glucuronide*	[33]
3	41.2	267	—	Quercetin-3-O-*β*-D-glucuronide methyl ester*	[7]
4	47.1	334	521.09094 [M + H]^+^/519.07715 [M − H]^−^	Ajugasterone C-20, 22-monoacetonide	[35]
5	52.6	256, 370	303.04901 [M + H]^+^/301.03473 [M − H]^−^	Quenterin*	[8]
18	53.2	255	754.25336 [M + NH_4_]^+^/735.21295 [M − H]^−^, 781.21869 [M + HCOOH-H]^−^	Helonioside B	[8, 31]
6	58.9	315	798.25769 [M + NH_4_]^+^/779.21729 [M − H]^−^, 825.22278 [M + HCOOH-H]^−^	Hydropiperoside	[8]
7	63.1	315	840.26898 [M + NH_4_]^+^/821.22845 [M − H]^−^, 867.23395 [M + HCOOH-H]^−^	Vanicoside C	[8, 36]
10	67.2	316	974.30463 [M + NH_4_]^+^/955.26428 [M − H]^−^, 477.12866 [M−2H]^2−^	Vanicoside B	[8]
11	69.0	317	1016.31396 [M + NH_4_]^+^/997.27496 [M − H]^−^, 498.13388 [M−2H]^2−^	Vanicoside F	[8]
12	71.3	317	—	Unknown	—

^a^RT, NI, and PI stand for retention time, negative-ion mode, and positive-ion mode, respectively.

*Identified by comparison with reference compounds.

**Table 6:** Profile-efficacy relationship results obtained from hot plate test. Values having good interrelationship with analgesic activity are marked with “∗.”

RT (min)	Peak no.	15 min			30 min			60 min			90 min		
		Low dose	Middle dose	High dose	Low dose	Middle dose	High dose	Low dose	Middle dose	High dose	Low dose	Middle dose	High dose
11.8	14	−0.0404	0.1283	0.2049	0.0940	0.1624	−0.0447	0.0590	0.1952	0.2519	0.1711	0.3286*	0.1137
13.2	1	0.0857	0.0489	0.3747*	0.1354*	0.1906*	0.2320*	0.2153*	0.4276*	0.5165*	0.1746*	0.3140*	0.2614*
15.5	15	0.1601	0.1702	0.3539*	0.1817*	0.1855*	0.2340*	0.1993*	0.2896*	0.3755*	0.2378*	0.4332*	0.2773*
19.0	16	0.1459	0.0306	0.3298*	0.1759*	0.2204*	0.3064*	0.2029*	0.3730*	0.5483*	0.2381*	0.3060*	0.3325*
22.4	17	0.1099	0.0888	0.3602*	0.1626*	0.1680*	0.3605*	0.2285*	0.3830*	0.5319*	0.2371*	0.3075*	0.3638*
36.0	2	0.0666	−0.0610	0.4049*	0.1638*	0.0740	0.2983*	0.2123*	0.4531*	0.3246*	0.1191*	0.1662*	0.1711*
41.2	3	0.1837	−0.0257	0.4001*	0.2147*	0.1798*	0.1144*	0.2114*	0.4546*	0.4503*	0.1505*	0.2473*	0.2735*
47.1	4	0.0520	0.3021	0.1349*	0.1036*	0.2062*	0.1751*	0.2946*	0.3538*	0.2276*	0.1753*	0.2591*	0.1621*
52.6	5	0.1055	0.0943	0.4455*	0.2260*	0.1811*	0.3304*	0.2477*	0.4484*	0.3656*	0.2298*	0.2839*	0.3481*
53.2	18	0.1377	0.0371	0.1936	0.2129	−0.0704	−0.2850	0.1779	0.2499	−0.1009	0.0957	0.1629	−0.1515
58.9	6	0.2282	0.2139	0.0404	0.2655	0.0551	−0.0249	0.1851	−0.0330	−0.2071	0.0013	−0.0475	−0.0565
63.1	7	0.2992	−0.0142	−0.0068	0.2516	0.0192	0.1552	0.1040	−0.0366	−0.0977	−0.0324	−0.0824	−0.0769
67.2	10	0.1522	0.4031	0.0618	0.2626	0.2584	−0.0409	0.1376	0.1952	−0.2190	0.0045	0.1306	−0.0006
69.0	11	0.2296	0.2589	0.0207	0.2253	0.1359	−0.0884	0.0637	0.1224	−0.2592	−0.0058	0.0768	0.0768
71.3	12	0.1103	0.0876	−0.1463	0.1254	0.0855	0.1594	0.0144	−0.0276	−0.1233	−0.1239	−0.0430	−0.0430

**Table 7:** Profile-efficacy relationship results obtained from writhing test. Values having positive correlation with analgesic activity are marked with “∗.”

RT (min)	Peak no.	Profile-efficacy relationship coefficients		
		Low dose	Middle dose	High dose
11.8	14	0.2102*	0.2433*	0.0400
13.2	1	0.2381*	0.2954*	0.3653*
15.5	15	0.3216*	0.3319*	0.0298*
19.0	16	0.0114*	0.1693*	0.3137*
22.4	17	0.1616*	0.1990*	0.3188*
36.0	2	0.0322*	0.2843*	0.4188*
41.2	3	0.1896*	0.2709*	0.2055*
47.1	4	−0.2428	−0.0293	0.0005
52.6	5	0.1407*	0.2076*	0.4099*
53.2	18	−0.0782	0.0255	−0.1666
58.9	6	−0.0283	−0.1021	0.0104
63.1	7	0.4290*	0.0076*	0.0109*
67.2	10	0.4757	−0.0693	0.0888
69.0	11	0.0402	0.0306	−0.0140
71.3	12	0.4342	−0.0696	0.1342

**Table 8:** Profile-efficacy relationship results obtained from ear edema test. Values having good correlation with anti-inflammation activity are marked with “∗.”

RT (min)	Peak no.	Profile-efficacy relationship coefficients	
		Low dose	High dose
11.8	14	0.3260*	0.3040*
13.2	1	0.3215*	0.3440*
15.5	15	0.1985*	0.1961*
19.0	16	0.3630*	0.3384*
22.4	17	0.1501*	0.2328*
36.0	2	0.2440*	0.3175*
41.2	3	0.3623*	0.3248*
47.1	4	0.0823	0.1042
52.6	5	0.2474*	0.3822*
53.2	18	0.2843	0.0516
58.9	6	−0.1626	0.1328
63.1	7	−0.2391	0.0412
67.2	10	−0.2808	0.0522
69.0	11	−0.1344	0.0215
71.3	12	−0.1831	0.0512

**Table 9:** Profile-efficacy relationship results obtained from vascular permeability test. Values having good correlation with anti-inflammation activity are marked with “∗.”

RT (min)	Peak no.	Profile-efficacy relationship coefficients		
		Low dose	Middle dose	High dose
11.8	14	−0.1636	0.0701	−0.1652
13.2	1	0.3345*	0.2735*	0.3172*
15.5	15	0.2514*	0.3288*	0.3057*
19.0	16	0.2673*	0.2132*	0.2904*
22.4	17	0.2468*	0.1859*	0.3830*
36.0	2	0.2689*	0.2850*	0.4443*
41.2	3	0.3507*	0.3015*	0.5092*
47.1	4	0.0342	0.0842	0.1553
52.6	5	0.0523	0.3550*	0.3630*
53.2	18	−0.2720	−0.3703	−0.2032
58.9	6	−0.1636	0.1776	0.2480
63.1	7	−0.0946	0.2732	0.1672
67.2	10	0.0519	0.0538	0.0353
69.0	11	−0.0921	−0.1866	−0.1918
71.3	12	0.0272	0.3015	0.1249
